# RDBSB: a database for catalytic bioparts with experimental evidence

**DOI:** 10.1093/nar/gkae844

**Published:** 2024-10-03

**Authors:** Wan Liu, Pingping Wang, Xinhao Zhuang, Yunchao Ling, Haiyan Liu, Sheng Wang, Haihan Yu, Liangxiao Ma, Yuguo Jiang, Guoping Zhao, Xing Yan, Zhihua Zhou, Guoqing Zhang

**Affiliations:** National Genomics Data Center & Bio-Med Big Data Center, CAS Key Laboratory of Computational Biology, Shanghai Institute of Nutrition and Health, University of Chinese Academy of Sciences, Chinese Academy of Sciences, 320 Yueyang Road, Shanghai 200031, China; CAS-Key Laboratory of Synthetic Biology, CAS Center for Excellence in Molecular Plant Sciences, Chinese Academy of Sciences, 300 Feng Lin Road, Shanghai 200032, China; National Genomics Data Center & Bio-Med Big Data Center, CAS Key Laboratory of Computational Biology, Shanghai Institute of Nutrition and Health, University of Chinese Academy of Sciences, Chinese Academy of Sciences, 320 Yueyang Road, Shanghai 200031, China; National Genomics Data Center & Bio-Med Big Data Center, CAS Key Laboratory of Computational Biology, Shanghai Institute of Nutrition and Health, University of Chinese Academy of Sciences, Chinese Academy of Sciences, 320 Yueyang Road, Shanghai 200031, China; School of Life Sciences, University of Science and Technology of China, 443 Huangshan Road, Hefei, Anhui 230026, China; Shanghai Zelixir Biotech Company Ltd., 4/F, Youyue Building, No. 298, Xiangke Road, Pudong New District, Shanghai 200030, China; School of Life Sciences, University of Science and Technology of China, 443 Huangshan Road, Hefei, Anhui 230026, China; National Genomics Data Center & Bio-Med Big Data Center, CAS Key Laboratory of Computational Biology, Shanghai Institute of Nutrition and Health, University of Chinese Academy of Sciences, Chinese Academy of Sciences, 320 Yueyang Road, Shanghai 200031, China; Shanghai Key Laboratory of Plant Functional Genomics and Resources, Shanghai Chenshan Botanical Garden, and Chenshan Science Research Center, CAS Center for Excellence in Molecular Plant Sciences (CEMPS), Chinese Academy of Sciences (CAS), 3888 Chenhua Road, Shanghai 201602, China; National Genomics Data Center & Bio-Med Big Data Center, CAS Key Laboratory of Computational Biology, Shanghai Institute of Nutrition and Health, University of Chinese Academy of Sciences, Chinese Academy of Sciences, 320 Yueyang Road, Shanghai 200031, China; School of Life Science, Hangzhou Institute for Advanced Study, University of Chinese Academy of Sciences, 1 Sub-lane Xiangshan, Hangzhou 310024, China; CAS-Key Laboratory of Synthetic Biology, CAS Center for Excellence in Molecular Plant Sciences, Chinese Academy of Sciences, 300 Feng Lin Road, Shanghai 200032, China; CAS-Key Laboratory of Synthetic Biology, CAS Center for Excellence in Molecular Plant Sciences, Chinese Academy of Sciences, 300 Feng Lin Road, Shanghai 200032, China; National Genomics Data Center & Bio-Med Big Data Center, CAS Key Laboratory of Computational Biology, Shanghai Institute of Nutrition and Health, University of Chinese Academy of Sciences, Chinese Academy of Sciences, 320 Yueyang Road, Shanghai 200031, China

## Abstract

Catalytic bioparts are fundamental to the design, construction and optimization of biological systems for specific metabolic pathways. However, the functional characterization information of these bioparts is frequently dispersed across multiple databases and literature sources, posing significant challenges to the effective design and optimization of specific chassis or cell factories. We developed the Registry and Database of Bioparts for Synthetic Biology (RDBSB), a comprehensive resource encompassing 83 193 curated catalytic bioparts with experimental evidences. RDBSB offers their detailed qualitative and quantitative catalytic information, including critical parameters such as activities, substrates, optimal pH and temperature, and chassis specificity. The platform features an interactive search engine, visualization tools and analysis utilities such as biopart finder, structure prediction and pathway design tools. Additionally, RDBSB promotes community engagement through a catalytic bioparts submission system to facilitate rapid data sharing and utilization. To date, RDBSB has supported the contribution of >1000 catalytic bioparts. We anticipate that the database will significantly enhance the resources available for pathway design in synthetic biology and serve essential tools for researchers. RDBSB is freely available at https://www.biosino.org/rdbsb/.

## Graphical Abstract

**Graphical Abstract:**
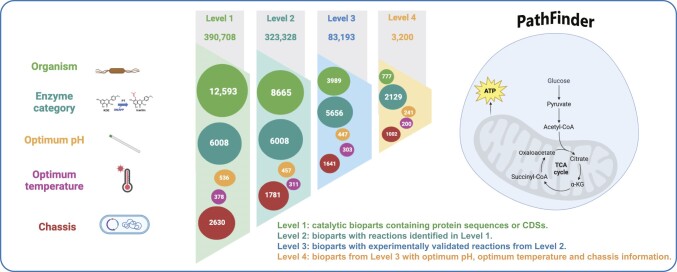

## Introduction

A primary goal of synthetic biology is the construction of artificial biological systems with novel functions archived through bottom-up forward engineering principles (1). Bioparts, as the fundamental building blocks, are indispensable and serve as the cornerstone of synthetic biology research and application (2,3). Catalytic bioparts refer to not only enzymes or other biomolecules that can catalyze biochemical reactions, but also their performance data and nuclear acid sequences in specific chassis (4,5). The selection and utilization of catalytic bioparts directly influence the efficiency and specificity of biosynthetic systems (6). By precise designing and assembling various catalytic bioparts, novel biological systems can be created for the production of valuable chemicals, pharmaceuticals, biofuels and other products (7–9).

Constructing biosynthetic systems needs to assemble target biosynthetic pathways within specific chassis in a ‘plug-and-play’ manner based on standardized catalytic bioparts (10,11). Thus, a reliable database of experimentally validated catalytic bioparts is essential for providing the resources required to design and optimize these biosynthetic systems. The Registry of Standard Biological Parts includes over 300 catalytic bioparts developed to address specific challenges in iGEM competition (12). While databases such as Swiss-Prot, MetaCyc, KEGG, BRENDA, CAZy and GTDB focus on curated protein (13), biosynthetic pathways (14,15) or enzyme (16–18) in non-engineered organism, they lack crucial information on the performance of catalytic biopart within chassis, which is vital for synthetic biology applications.

To address the gap, we developed the Registry and Database of Bioparts for Synthetic Biology (RDBSB), which contained 83 193 experimentally validated catalytic bioparts. We have also systematically reviewed and compiled comprehensive information for the application of catalytic bioparts, including optimal pH and temperature, as well as chassis. RDBSB provides an interactive data search engine and visualization interface for the convenience of users. Additionally, it offers a suite of efficient tools, including catalytic biopart finders such as BiopartFinder and MapView, structure prediction tools such as AlphaFold (19) and PVQD (20) and pathway design tool PathFinder, all of which provide robust support for discovering and utilizing catalytic biopart data. Furthermore, we have designed an effective submission system for sharing of catalytic biopart information. RDBSB is freely available at https://www.biosino.org/rdbsb/.

## Materials and methods

**Figure 1.**
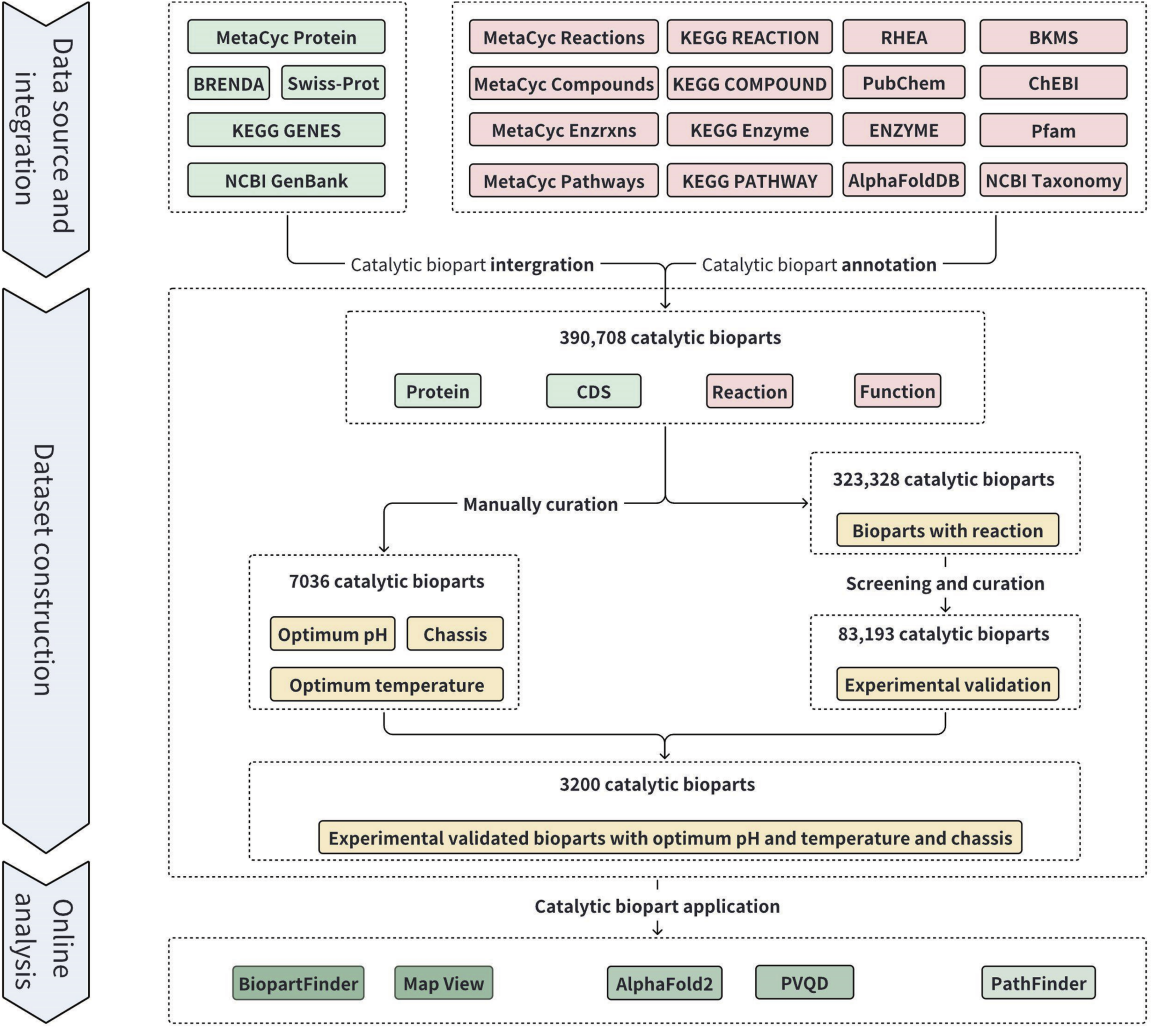
The overview of RDBSB workflow. This workflow contains three steps, including data source and integration, dataset construction and online analysis.

The methodology for developing RDBSB is illustrated in Figure 1.

### Data source and integration

MetaCyc Protein, BRENDA, Swiss-Prot, KEGG GENES and NCBI GenBank served as the primary resources for integrating catalytic bioparts, providing crucial information on proteins, coding sequences (CDSs), reactions and functions (13–15,17,21). Reactions were sourced from MetaCyc Reactions, MetaCyc Enzrxns, KEGG REACTION, RHEA and BKMS (22,23), while substrate and product annotations were derived from MetaCyc Compounds, KEGG COMPOUND, PubChem and ChEBI (24,25). KEGG Enzyme, ENZYME, Pfam, MetaCyc pathways, KEGG PATHWAY, AlphaFoldDB and NCBI Taxonomy were used to annotate the enzyme, domain, pathway, structure and organism associated with each biopart (26–29) (Figure 1). Following data integration and deduplication, 390 708 catalytic bioparts were extracted.

### The screening and curation of experimental validated reaction

All bioparts were screened for experimental validation based on reaction information. First, bioparts lacking reaction information in any source database were excluded, leaving 323 328 catalytic bioparts associated with reactions for further analysis (13–15,17). Second, bioparts marked with the ‘Evidence at protein level’ designation by Swiss-Prot were immediately classified as having experimental validation. Third, the remaining bioparts were evaluated for experimental evidence based on the presence of literature-supported reaction information in any of the source databases. Additionally, the reactions catalyzed by cytochrome P450 (P450) and glycosyltransferase GT1 (GT1) family enzymes were directly curated from the literature. In total, 83 193 of these bioparts were confirmed as experimentally validated.

### The curation of experimental condition

We employ a ‘two-curator, one-reviewer’ method for manual review. In this process, two curators independently conduct assessments. If their conclusions align, the result is immediately confirmed. However, if discrepancies arise, a reviewer is brought in to facilitate discussions between the curators, and the final outcome is based on the consensus reached. For chassis review, enzyme method descriptions from BRENDA were curated using this approach, leading to the standardization of 2630 chassis description. Similarly, to determine the optimum temperature and pH for catalytic bioparts, literatures from Swiss-Prot were curated and structured into 2636 temperature records for 3049 bioparts and 4325 pH records for 8637 bioparts.

Additionally, RDBSB incorporated 10 535 structured optimum pH records and 9452 optimum temperature records from BRENDA, along with 590 optimum pH records and 319 optimum temperature records from MetaCyc. During the curation and incorporation process, pH and temperature values were standardized to ranges of 0–14 for pH and 0–125°C for temperature. As a result, 27 789 bioparts were associated with pH or temperature or chassis information.

### The curation of P450 and GT1 family enzymes

Given the critical roles of P450 in oxygenation and GT1 in glycosylation of natural products, our curation efforts were specifically focused on these bioparts (21,22). To identify potential P450 and GT1 bioparts, we constructed a hidden Markov model library based on sequence profiles using HMMER (version 3.1b2) (30). The profile of GT1 family was obtained from dbCAN (version 8) (31), and the profile of P450 sourced from Pfam (version 32) (27). We then mined potential P450 and GT1 protein sequences from Swiss-Prot and the NCBI Non-Redundant Protein Sequence Database (NR, January 2020 download) (32) using hmmscan (pipe-0.0.1 r2, with parameters: e-value 1e−5, coverage 0.4) (33). For the proteins identified in Swiss-Prot, we reviewed associated biochemical reactions, kinetic parameters and evidence levels. To further refine the dataset, we filtered the literature associated with proteins from NR using keywords such as SDS–PAGE, HPLC, LC–MS, gas chromatography, GC–MS and TLC, ultimately selecting 276 P450 papers and 224 GT1 papers for detailed curation.

In curating P450 and GT1 bioparts, we aimed for comprehensive coverage by adhering to previously established curation methods. To address issues related to incomplete or non-standardized reactions in the literature, we supplemented the reactions with established chemical principles, reconstructed structural formulas for substrates and products, and ensured accurate naming and database ID assignment. Additionally, substrates and products were standardized via PubChem and ChEBI. For entries that could not be matched, structures were generated in SMILES format using ChemDraw (34), ensuring consistency and reliability across our curated data.

### Database implementation

The RDBSB dataset can be visualized through web browsers and was developed using the Spring Boot framework (https://spring.io/projects/spring-boot), with core JavaScript libraries including jQuery (https://jquery.com/) and Echarts (https://echarts.apache.org/). Elasticsearch (https://www.elastic.co) is utilized to optimize data retrieval performance. Additionally, we used NCBI BLAST (v.2.13.0+) (35) for sequence alignment, AlphaFold2 (v2.0.1) (19) for tertiary structure prediction and PVQD (20) for multidimensional conformational prediction of catalytic biopart. The 3D structures predicted by AlphaFold and PVQD were displayed by Mol* Viewer (36).

### PathFinder development

PathFinder is a tool we developed to facilitate route design from substrate to product via possible pathways. We construct a directed graph *G*$ = ( {V,E} )$, where the vertex set $V$ represents individual substrates, reaction and individual products, while the edge set $E$ represents the chemical reactions or combination relationships between these vertices. If a reaction $r$ transforms substrate $S$ into product $P$, the edge $e = ( {S,P} )$ represents this transformation. To accelerate query speed, we excluded common compounds such as H_2_O, ATP, cofactor NADPH and coenzyme CoA from the network during graph construction. After building the graph for the RDBSB dataset, we use the allShortestPaths algorithm in Neo4j for path searching (https://neo4j.com/docs/cypher-manual/current/patterns/reference/), which efficiently traverses the shortest path in a directed graph with weighted edges.

## Results

### Overview of RDBSB

The integrity of biopart information is classified into four levels:

- Level 1 refers to catalytic bioparts that contain protein sequences or CDSs.
- Level 2 includes bioparts from Level 1 that have associated reactions.
- Level 3 comprises bioparts from Level 2 with experimentally validated reactions.
- Level 4 consists of bioparts from Level 3 that also have optimum pH, optimum temperature and chassis information.

In total, 390 708 catalytic bioparts were integrated from various database sources, including 83 193 that have been experimentally validated, which far exceed the coverage of other enzyme databases such as BRENDA in terms of experimentally validated catalytic bioparts (17). Of these, 3200 experimentally validated catalytic bioparts include curated data on optimum temperature, optimum pH and chassis (Figure 1).

**Figure 2.**
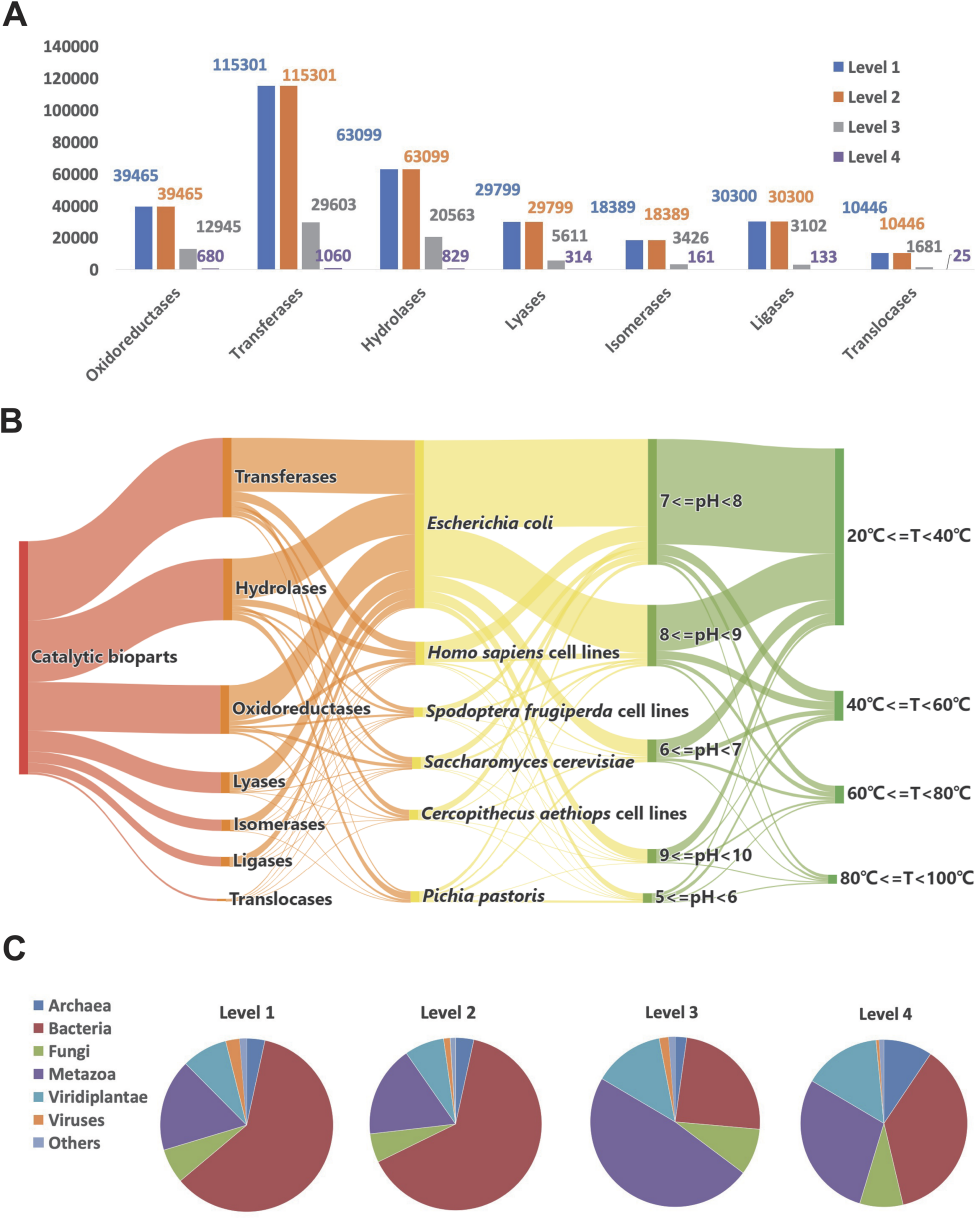
Data overview of RDBSB. (**A**) Distribution of catalytic bioparts across enzyme categories classified according to ENZYME database. Level 1: catalytic bioparts containing protein sequences or CDSs; Level 2: bioparts with reactions identified in Level 1; Level 3: bioparts with experimentally validated reactions from Level 2; and Level 4: bioparts from Level 3 with optimum pH, optimum temperature and chassis information. (**B**) Distribution of experimentally validated bioparts across enzyme category, chassis, optimum pH and temperature. (**C**) Distribution of source organisms at varying levels of information integrity categorized according to NCBI Taxonomy.

The top three enzyme categories of bioparts are transferases, hydrolases and oxidoreductases (Figure 2A). The optimum temperature and pH for these bioparts are predominantly within the ranges of 20–40°C and pH 6–9, respectively (Figure 2B), which align with the optimal conditions for commonly used laboratory chassis, such as *Saccharomyces cerevisiae, Escherichia coli, Bacillus subtilis, Corynebacterium glutamicum* and *Nicotiana tabacum*. Most source organisms of these bioparts belong to the Bacteria, Metazoa and Viridiplantae groups (Figure 2C).

### Web user interface

RDBSB offers a user-friendly interface with versatile tools to visualize and retrieve catalytic bioparts for cell factory construction. Key features include (i) a quick navigation tool for locating bioparts with varying levels of information integrity; (ii) a keyword search function for retrieving bioparts by names, identifiers of public resources, compounds, chassis and pathways; (iii) filtering options to combine different experimental conditions to find suitable biopart, such as chassis, optimum pH and temperature and integrity level; and (iv) BiopartFinder, which enables amino acid sequence searches to identify relevant bioparts and potential alternatives or reaction conditions for pathway design in chassis.

**Figure 3.**
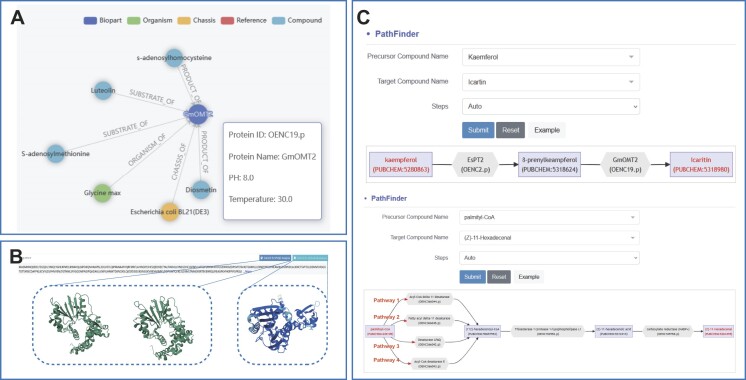
Online analysis served by RDBSB. (**A**) MapView example of GmOMT2. The MapView provides on related organism, chassis, reference and compound information of a specific biopart. Hovering over the biopart name displays brief details. (**B**) User interface for 3D conformation and structure analysis of GmOMT2 by PVQD and AlphaFold. Two conformations of GmOMT2 generated by PVQD are displayed in green and the structure of GmOMT2 predicted by AlphaFold is shown in blue. (**C**) PathFinder application showcasing examples of artificial biosynthetic pathway: one for drug icaritin pathways in yeast, and another for insect sex pheromones pathways in yeast.

On the detailed page for each biopart, users can access comprehensive information on catalytic functions, supported by literature references to ensure accuracy and reliability. The page also displays alternative bioparts involved in the same reaction, aiding in cell factory construction and optimization. The MapView tool allows users to interact and visually explore relationships between bioparts, chassis, organisms, compounds and literature (Figure 3A). At the bottom of the page, users can access amino acid and CDSs for laboratory use, along with structure prediction tools such as AlphaFold and PVQD. AlphaFold is used to predict precise structures for catalytic bioparts (19), while PVQD offers predictions of various conformations, providing structural insights for designing more efficient and specific bioparts (20) (Figure 3B).

### Application of RDBSB for natural products synthetic biology research

The construction of biosynthetic pathways to produce value-added compounds is a primary objective in metabolic engineering and synthetic biology (4,37). With over 80 000 experimentally validated catalytic bioparts available, it is now possible to identify suitable biosynthetic pathways for desired natural products or even design artificial pathways to synthesize target compounds with previously unknown pathways. We utilize PathFinder for pathway design, as demonstrated by two examples (Figure 3C).

### Biosynthesis of icaritin

Icaritin is a prenylflavonoid present in the Chinese herbal medicinal plants *Epimedium* spp., and is currently used in the treatment of advanced hepatocellular carcinoma (38). Structurally, icaritin was synthetized from kaempferol via two steps, the prenylation at C8 and the methylation at C4′-OH of kaempferol. Since both prenyltransferase and methyltransferase are superfamily enzymes and their function and sequence relationship are not clear, it is very challenging to screen suitable prenyltransferase and methyltransferase with desired function from certain plant to fulfill this pathway. We then test PathFinder for the designing of this pathway, when kaempferol and icaritin were input as the substrate and product, respectively, PathFinder designed a biosynthetic pathway for icaritin. Kaempferol was first converted to 8-prenylkaempferol by EsPT2 and then 8-prenylkaempferol was converted to icaritin by GmOMT2. However, directly reconstructing this pathway in yeast cytoplasm led to no icaritin production. As deposited in RDBSB, the cytoplasmic pH of yeast is typically ∼5.5–7, while the biopart GmOMT2 lost function at pH < 6.5; hence, the incompatibility between the suitable pH of biopart and the cytosol pH of yeast chassis led to the failure of *de novo* icaritin biosynthesis. Complete biosynthesis of icaritin has been successfully achieved by reconstructing this pathway through relocating GmOMT2 into the mitochondria with relatively higher pH (∼7.5) of yeast (39).

### Biosynthesis of *cis-*11-hexadecenal

Another example for pathway design is the biosynthesis of *cis-*11-hexadecenal (Z11-16:Ald), a key component of sex pheromones in several notorious agricultural pests. Z11-16:Ald was taken as a biosynthesis target to broaden field application at low cost. However, the natural biosynthetic pathway of Z11-16:Ald in *Helicoverpa armigera* remains largely unknown. A proposed biosynthetic pathway was from palmitoyl-CoA through three-step conversion: palmitoyl-CoA was first converted to (11*Z*)-hexadecenoyl-CoA by fatty acyl desaturase, and then (*Z*)-11-hexadecenol was generated by fatty acyl reductase, which was then converted to Z11-16:Ald by alcohol oxidase, while the fatty acyl desaturase and alcohol oxidase remain unknown. When palmitoyl-CoA and Z11-16:Ald were input as the substrate and product, respectively, PathFinder proposed four possible pathways: palmitoyl-CoA was first converted to (11*Z*)-hexadecenoyl-CoA by desaturase from four different sources, and then thioesterase releases the CoA of (11*Z*)-hexadecenoyl-CoA to generate the corresponding hexadecanoate product, which was subsequently converted to Z11-16:Ald by carboxylic acid reductase. Notably, all these four pathways were ‘unnatural’; the route from palmitoyl-CoA to Z11-16:Ald was different from the proposed biosynthetic pathway in *H. armigera*. Results indicated that PathFinder could design artificial pathways to synthesize target compounds with unknown pathways. All the designed pathways were successfully reconstructed in budding yeast to construct cell factories; two of the designed pathways (pathways 1 and 2) resulted in the successful production of the target Z11-16:Ald, with pathway 1 giving the highest production level (40).

### Application of RDBSB for the deposition of catalytic bioparts

RDBSB supports the online submission of catalytic bioparts, enabling researchers to submit according to the provided guidelines. Upon review, RDBSB assigns accession numbers to the bioparts. Additionally, RDBSB facilitates the deposition and sharing of related plasmids and strains, which can be made available to the research community upon request. Since its launch in 2019, RDBSB has supported the publication of over 200 catalytic bioparts in high-quality journals and has distributed numerous bioparts to the synthetic biology community (39–45). Through this system, manually curated 142 P450 bioparts and 120 GT1 bioparts in this study have been submitted to RDBSB.

## Discussion

RDBSB is a comprehensive platform designed for the collection, storage and sharing of detailed qualitative and quantitative data on catalytic bioparts. It also offers practical tools for applying catalytic bioparts in natural product synthesis, contributing to the advancement of synthetic biology. To the best of our knowledge, we have aggregated as many catalytic bioparts as possible from public resources, with over 80 000 supported by experimental evidence through manual curation and literature mining. RDBSB emphasizes the collection and curation of experimental conditions, including optimum pH and temperature, and compatible chassis. Additionally, our system supports and encourages the submission of new catalytic bioparts, continually enriching the repository with experimental validated data.

To date, our database has received >500 000 visits. Its impact is becoming increasingly evident, with over 200 catalytic bioparts published in well-known journals such as *Green Chemistry* and *Science Bulletin*. We also provide tools such as PathFinder for designing biosynthetic pathways, which have successfully replicated two recently reported synthetic pathways. Looking ahead, we aim to incorporate energy requirements into pathway optimization. By introducing an energy threshold ${{E}_{{\rm thres}}}$, we hope to filter synthetic routes to identify those that are more efficient and energy-saving. This could enhance the economic feasibility of biosynthetic technologies and contribute to the development of green and sustainable chemistry.

In summary, RDBSB expands the resources available for synthetic biology research and applications, while offering tools for studying the functions of catalytic bioparts. We hope that RDBSB will become a valuable resource for synthetic biology and look forward to making further contributions to the field through ongoing development and collaboration.

## Data Availability

RDBSB is freely available at https://www.biosino.org/rdbsb/.
